# Advancing the chemotherapy of tuberculous meningitis: a consensus view

**DOI:** 10.1016/S1473-3099(24)00512-7

**Published:** 2024-09-26

**Authors:** Sean Wasserman, Joseph Donovan, Evelyne Kestelyn, James A. Watson, Robert E. Aarnoutse, James R. Barnacle, David R. Boulware, Felicia C. Chow, Fiona V. Cresswell, Angharad G. Davis, Kelly E. Dooley, Anthony A. Figaji, Diana M. Gibb, Julie Huynh, Darma Imran, Suzaan Marais, David B. Meya, Usha K. Misra, Manish Modi, Mihaja Raberahona, Ahmad Rizal Ganiem, Ursula K. Rohlwink, Rovina Ruslami, James A. Seddon, Keira H. Skolimowska, Regan S. Solomons, Cari J. Stek, Nguyen Thuy Thuong Thuong, Reinout van Crevel, Claire Whitaker, Guy E. Thwaites, Robert J. Wilkinson

**Affiliations:** 1 Centre for Infectious Diseases Research in Africa, Institute of Infectious Disease and Department of Medicine, University of Cape Town, Observatory 7925, Republic of South Africa; 2 St George’s, University of London, Cramner Terrace, London SW17 0RE, United Kingdom; 3 London School of Hygiene and Tropical Medicine, Keppel Street London WC1E 7HT United Kingdom; 4 Oxford University Clinical Research Unit, Ho Chi Minh City, 700 000 Vietnam; 5 Radboud University Medical Center, Nijmegen, Netherlands; 6 The Francis Crick Institute, London, NW1 1AT, United Kingdom; 7 Department of Infectious Diseases, Imperial College London, W12 0NN, United Kingdom; 8 Division of Infectious Diseases and International Medicine, Department of Medicine, University of Minnesota, Minneapolis, MN 55455, United States of America; 9 Departments of Neurology and Medicine (Infectious Diseases), University of California San Francisco, San Francisco, CA 94143, United States of America; 10 Infectious Diseases Institute, School of Medicine, College of Health Sciences, Makerere University, Kampala, Uganda; 11 HIV Interventions, MRC/UVRI-LSHTM Uganda Research Unit, Entebbe, Uganda; 12 Global Health and Infection, Brighton and Sussex Medical School, Brighton, BN1 9PX UK; 13 Division of Infectious Diseases, Vanderbilt University Medical Center, Nashville, TN 37232, United States of America; 14 Division of Neurosurgery, Department of Surgery, Neuroscience Institute, University of Cape Town, Observatory 7925, Republic of South Africa; 15 Medical Research Council Clinical Trials Unit, London, WC1V 6LJ United Kingdom; 16 Centre for Tropical Medicine and Global Health, Nuffield Department of Medicine, Oxford OX1 4BH United Kingdom; 17 Dr. Cipto Mangunkusumo Hospital, Jakarta, Indonesia; 18 Division of Neurology, Department of Medicine, University of Cape Town, Observatory 7925, Republic of South Africa; 19 Neurology Research Group, Neuroscience Institute, University of Cape Town, Observatory 7925, Republic of South Africa; 20 Department of Medicine, School of Medicine, College of Health Sciences, Makerere University, Kampala, Uganda; 21 Medical College, Vivekanand Polyclinic and Institute of Medical Sciences and Apollo Medics Super Speciality Hospital, Lucknow, India; 22 Postgraduate Institute of Medical Education and Research, Chandigarh, PIN 160012 India; 23 University Hospital Joseph Raseta Befelatanana, Antananarivo, Madagascar; 24 Department of Neurology, Faculty of Medicine Universitas Padjadjaran / Hasan Sadikin Hospital, Bandung, Indonesia; 25 Department of Biomedical Sciences, Faculty of Medicine, Universitas Padjadjaran, Bandung, Indonesia; 26 Department of Paediatrics and Child Health, Faculty of Medicine and Health Sciences, Stellenbosch University, Tygerberg 7505, Republic of South Africa

## Abstract

Tuberculous meningitis (TBM) causes death or disability in approximately 50% of those affected and kills approximately 78200 adults every year. Antimicrobial treatment is based on regimens used for pulmonary tuberculosis which overlooks important differences between lung and brain drug distributions. TBM has a profound inflammatory component, yet only adjunctive corticosteroids have shown clear benefit. There is an active pipeline of new antitubercular drugs, and the advent of biological agents targeted at specific inflammatory pathways promise a new era of improved TBM treatment and outcomes. Yet, to date, TBM trials have been relatively small, underpowered, heterogeneous, poorly generalizable, and have had little impact on policy and practice. Progress is slow, and a new approach is required. Here, a global consortium of TBM researchers articulate a coordinated, definitive way ahead via globally conducted clinical trials of novel drugs and regimens to advance treatment and improve outcomes from this life-threatening infection.

## Introduction

Tuberculous meningitis (TBM) is the most severe form of tuberculosis, causing death or disability in 50% of those affected^1^. An estimated 78,200 adult deaths result from TBM annually, with 35% of these in people living with HIV/AIDS (PWH). Despite such poor patient outcomes, little progress has been made over the last 40 years to identify evidence-based therapies and strategies that reduce death and disability from TBM; the exception being trials demonstrating that adjunctive corticosteroids reduce death in HIV-negative adults and children with TBM^2,3^.

Randomised controlled trials (RCT) in TBM are urgently required. This urgency comes at a time of opportunity for improving patient outcomes: the pipeline of new antitubercular drugs is more active than it has been for 50 years, and the advent of biological agents targeted at specific inflammatory molecules or pathways (e.g. TNF), both promise a new era of improved TBM therapy. Yet, to date, TBM trials have been relatively small, underpowered, heterogeneous, poorly generalisable, and have had little impact on policy. Progress is slow, and a new approach is due.

Recognising TBM to be a neglected form of tuberculosis, the Tuberculous Meningitis International Research Consortium was established in 2009. The consortium has contributed several reviews on aspects of epidemiology, pathogenesis, diagnosis, and management of TBM^4–18^. Membership includes over 100 researchers and TBM trial centres in India, Indonesia, Madagascar, South Africa, Uganda, and Vietnam. At consortium meetings in Oxford (2022) and in Cape Town (2023), a new approach to TBM trials and therapeutic development was conceived. A working group was formed, seeking to reach consensus agreement on the design, execution, and funding of future TBM clinical trials. This personal view summarises current knowledge on the therapeutic landscape for TBM, articulates a consensus view on research priorities, and sets out recommendations to accelerate improvements in TBM outcomes through globally conducted clinical trials.

## Therapeutic goals

### Effectively control Mycobacterium tuberculosis

TBM became a treatable disease in the 1940s with the discovery of the first antitubercular drugs, streptomycin and para-aminosalicylic acid. The bacterial killing induced by these two drugs reduced mortality from 100% to around 70%^19^. However, whilst bacterial killing is necessary for survival from TBM, the correlation between increased bacterial killing and better clinical outcomes has been elusive. This may be due to the difficulties of measuring killing with relatively few bacteria in cerebrospinal fluid (CSF), and the confounding influence of inflammation upon outcome. Clinical studies of TBM caused by isoniazid-resistant bacteria showed that resistance was associated with reduced times to CSF sterility, which in turn was associated with worse outcomes, especially in PWH^20^. However, clinical trials of ‘optimised’ antitubercular regimens that result in greater brain drug exposures have had mixed results. A trial of higher dose rifampicin (13 mg/kg given intravenously) confirmed higher drug exposure and documented increased survival^21^, but a much larger trial did not show any benefit of adding higher dose rifampicin (15 mg/kg orally) and levofloxacin to standard therapy unless disease was caused by isoniazid-resistant bacteria^22^. Nevertheless, despite equivocal findings of previous studies, improved brain penetration and thus faster bacterial killing may lead to better outcomes. This motivates testing new and potentially more active antitubercular drugs for TBM, and mandates associated pharmacometric studies that will enable better understanding of the complex relationship between drug exposure, bacterial killing, and clinical outcome^23^.

### Control host inflammation

Excessive host inflammation contributes to death and disability from TBM. Whilst limited evidence exists to guide adjunctive therapies, a landmark trial in Vietnam demonstrated that 6-8 weeks adjunctive dexamethasone reduced 9-month mortality in a predominantly HIV-negative group of adults and adolescents with TBM, though with no impact on disability^3^. However, current doses of corticosteroids may be insufficient to prevent and reduce host inflammation in all TBM patients, particularly in PWH^24^ and in the context of high dose rifampicin which increases corticosteroid clearance^25^. The recently published ACT-HIV trial (dexamethasone for TBM in PWH) from Vietnam and Indonesia did not conclusively establish a benefit of dexamethasone on survival in PWH^26^. In paediatric TBM, corticosteroids have also shown clinical benefit^27^. However, more targeted approaches to reducing tissue damaging host responses are emerging as understanding of TBM immunopathology deepens.

### Prevent and manage secondary neurological complications

Neurological complications majorly contribute to poor outcome from TBM. Most manifestations, including raised intracranial pressure, hydrocephalus, cerebral infarction, paradoxical reactions, and seizures, are a direct consequence of intracranial inflammation, emphasising the need to prioritise evaluation of more effective anti-inflammatory therapies. However, evidence-based interventions for adjunctive neurocritical care in TBM are also needed^28^. Trials defining the management of these important complications have never been conducted. This is partly because of resource constraints in high burden settings, for example limited access to expertise for ventricular drainage, and because of substantial challenges in trial design and implementation.

## Antitubercular chemotherapy

### General considerations

Current antitubercular chemotherapy for TBM remains based on that used in pulmonary tuberculosis and does not account for distinct disease characteristics in TBM that require specific therapeutic considerations. Use of the present regimen for TBM is not based on bespoke trials, rather on progress made in the derivation of ‘short course’ regimens for pulmonary tuberculosis. In contrast with pulmonary tuberculosis, where overall mortality is relatively low and the treatment goal is long-term relapse-free cure, TBM therapy must reduce early mortality and longer-term neurological disability. A primary consideration for TBM treatment is to select effective antitubercular drugs that rapidly achieve therapeutic concentrations at the site of disease. Drug penetration into brain tissue is key: CSF drug concentrations are often used but are an indirect and suboptimal measure of brain tissue penetration. There is limited pharmacometric evidence to support the composition, doses, or duration of the current standard of care for TBM. Other characteristics of an ideal TBM regimen include activity against drug-resistant *M. tuberculosis* strains, particularly isoniazid-resistant *M. tuberculosis*, which is common but infrequently detected in TBM; a low propensity for serious adverse reactions that may lead to premature discontinuation; and comprising drugs that can be dosed in neurocritical illness and that are accessible in high burden settings.

### Selection of new antitubercular drugs and regimens

Ongoing and recently completed unpublished trials are shown in Table 1. All actively recruiting phase 3 trials of antitubercular therapy are investigating high-dose rifampicin (35 mg/kg), often in combination with linezolid; several are powered to demonstrate reductions in mortality. Higher dose isoniazid is also being evaluated. Doses and composition of these experimental regimens were selected based on pharmacokinetic (PK)/pharmacodynamic (PD) data from patients with pulmonary tuberculosis (using sputum culture conversion as the efficacy measure) and small trials in TBM showing inconsistent effects on clinical outcome. Nevertheless, these ongoing studies will provide high-quality evidence to inform treatment guidelines. Future trials should avoid repeating evaluation of these regimens (which may become standard of care and serve as a control in future trials, if successful) and take different approaches to regimen selection.

Given the inability to perform frequent direct brain sampling in patients with TBM and the limitations of using CSF as a proxy for site of disease drug exposure, pre-clinical disease models are a promising strategy to optimise and select new drugs to enter TBM trials^29^. A translational TBM model should recapitulate key elements of human disease, including similar time course and clinical manifestations, typical histopathology, and compatible bacterial load and distribution. Importantly, TBM models should employ dosing schedules that are used in patients^30^. These features enable better predictive ability and may serve as potential efficacy markers. Animal models enable estimation of drug penetration within any CNS compartment at human-equivalent doses (determined by approximating plasma drug exposures from patients), which can be extrapolated to inform regimen design for trials. The underlying hypothesis is that rapid attainment of maximal effect exposures for a potent antitubercular agent at the site of disease in a relevant TBM model may translate into clinical efficacy. A major limitation of TBM models (and clinical trials) is lack of a predictive PD marker for treatment response. Application of efficacy measures from pulmonary tuberculosis, such as decline in bacterial load, do not necessarily predict clinical treatment response in TBM. This may lead to incorrect conclusions around clinical effectiveness of new drugs, with a risk of down-selecting potentially good candidates or promoting drugs that may not perform well in patients. Other limitations of preclinical models in TBM include altered oral bioavailability of combination regimens due to intolerance of high drug volumes during administration and differences in drug metabolism across species limiting evaluation of PK variability and drug-drug interactions.

**Figure 1:**
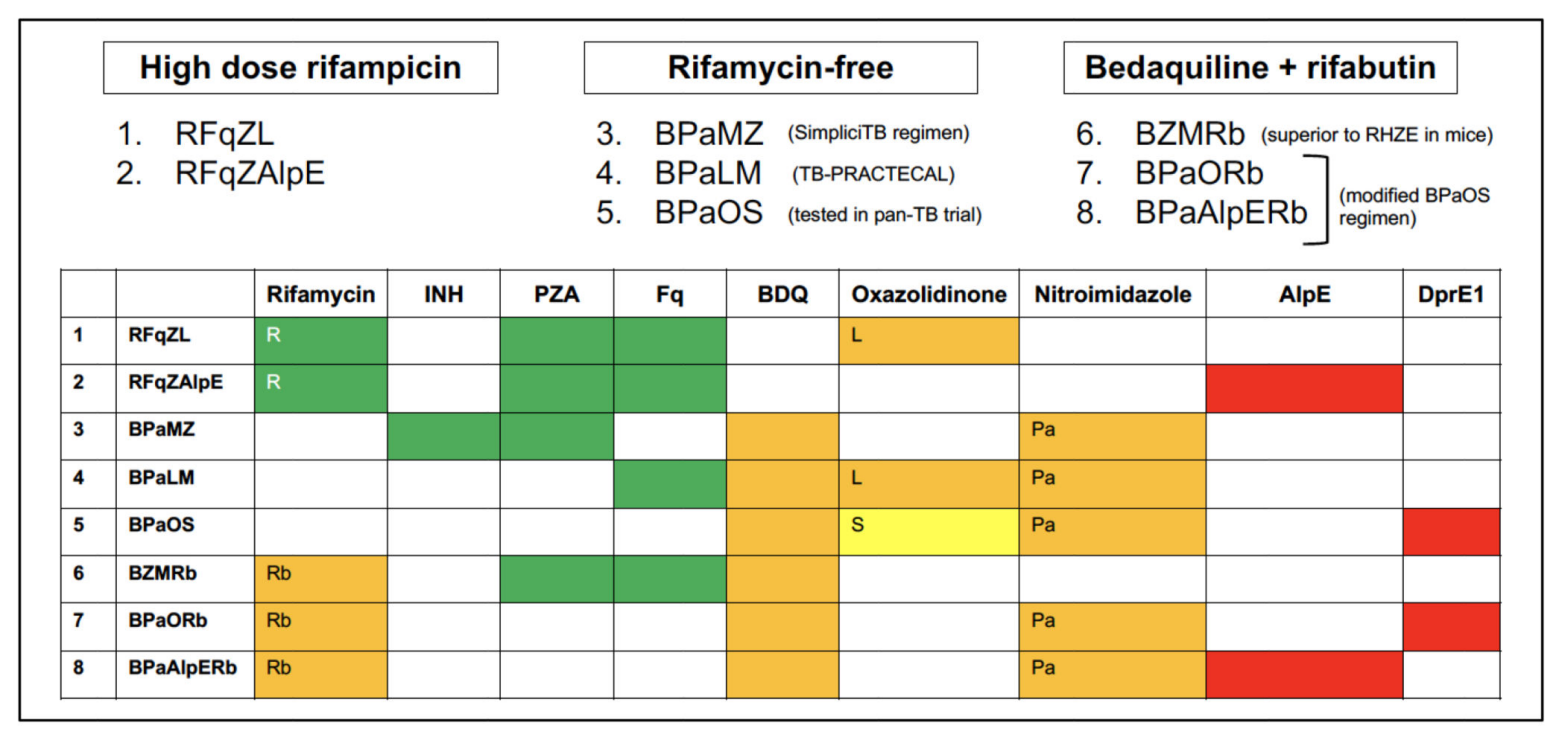
Potential regimens based on individual drug rankings. B = bedaquiline, H = isoniazid, L = linezolid, M = moxifloxacin, O = DprE1 inhibitor, Pa = pretomanid, Rb = rifabutin, S = sutezolid, AlpE = alpibectir/ethionamide, Z = pyrazinamide, R = rifampicin (high dose), Fq = fluoroquinolone, E = ethambutol. Coloured cells relate to individual drug scores on the ranking system, indicated in Table 3: green ≥ 12; orange 9 – 11; yellow < 9; red not currently manufactured.

Pre-clinical TBM models provide invaluable information about site-of-disease drug exposure but, because of their inherent limitations, TBM regimen selection needs to be informed by additional parameters aligned with the target regimen profile. These include antitubercular activity; observed clinical efficacy in TBM; safety and tolerability; potential for PK drug-drug interactions; and access in high burden settings. We propose a ranking system to select individual drugs for inclusion in experimental regimens for TBM (Table 2), using the approach adopted by the NIH ACTIV platform COVID-19 trials therapeutic agent selection committee (https://www.nih.gov/research-training/medical-research-initiatives/activ). New regimens of interest are constructed from individual agent rankings (Table 3), plus other considerations including *in vitro* synergy, combining different mechanisms of action, efficacy in pulmonary tuberculosis, and activity against drug resistant tuberculosis. Using this approach, three regimen categories emerge: (1) rifampicin-based; (2) bedaquiline-based; and (3) rifabutin-bedaquiline based (Figure 1).

### Towards rifampicin-free regimens for TBM

It remains possible that the ongoing trials, particularly if data are pooled, will establish the role of high-dose rifampicin in TBM. Future trials should therefore only plan evaluation of rifampicin-based regimens under a scenario where results of current studies are equivocal or indicate a need for further evaluation of high-dose rifampicin, possibly in combination with other agents. The development of an effective rifampicin-free regimen is a priority given the global threat of rifampicin-resistant tuberculosis. Drug-resistant TBM is associated with extremely high mortality and is under-recognised because of limited diagnostic sensitivity^31^. An individualised approach to treatment of TBM is therefore not possible and treatment regimens that cover drug-resistant disease are needed. An expanding evidence base supports this approach and creates conditions of potential equipoise to exclude rifampicin from treatment regimens in TBM. First, rifampicin concentrations at the site of disease in TBM animal models are variable and spatially heterogeneous^32^. In patients, rifampicin penetration into CSF is relatively poor compared to other antitubercular drugs^33–35^. Second, although several PK endpoint trials have suggested improved outcomes, rifampicin use has not conclusively led to survival benefit in randomised trials and pooled analyses, even at higher doses^22,34,36^. The perceived essentiality of rifampicin therapy for TBM is further undermined by case reports of treatment success with rifamycin-free regimens in patients with recognised rifampicin-resistant TBM. Third, rifamycin-free, bedaquiline-containing regimens perform better (cure and bactericidal) in mouse models of pulmonary tuberculosis and are highly successful in trials and clinical practice for patients with pulmonary tuberculosis, achieving comparable cure rates to standard rifampicin-based therapy for drug-susceptible tuberculosis^37–39^ In this context, investigating the effectiveness of bedaquiline (BDQ)-based regimens for TBM is a priority.

### Considerations for bedaquiline-based regimens

A highly lipophilic drug with extensive tissue distribution, bedaquiline has potential to concentrate within brain tissue. However, there are analytical challenges to quantifying bedaquiline in CNS compartments (binding to collection tubes, requirement for highly sensitive assays) and physiological barriers to CNS entry (extensive protein binding). Pre-clinical and clinical data on the CNS distribution of bedaquiline are limited; early investigations have shown measurable concentrations of total (bound and unbound fractions) drug in CSF from patients with pulmonary tuberculosis and relatively higher brain exposures in rodents^40–42^. The bedaquiline brain concentration associated with efficacy is unknown, but even limited exposure of this potent drug at the site of disease may provide benefit in TBM which is paucibacillary, particularly in combination with other effective agents. More detailed study of bedaquiline PK in representative animal disease models is a research priority.

Synergies with existing and new antitubercular agents that achieve high site of disease exposures may enhance potential efficacy of bedaquiline in TBM. Combining bedaquiline with pretomanid, linezolid, and moxifloxacin (BPaLM) has been highly successful for pulmonary tuberculosis and represents a promising regimen for TBM because of favourable PK characteristics of individual drugs. Similarly, the combination of BPaM plus pyrazinamide (BPaMZ), which was highly effective in the SimpliciTB trial for pulmonary tuberculosis, would likely achieve therapeutic concentrations in the CNS. However, hepatic toxicity from the combination of pyrazinamide plus pretomanid reduces enthusiasm for evaluation in TBM^43^. While fluoroquinolones have been extensively investigated and there is accumulating experience with linezolid in TBM, human pharmacometric data are limited for pretomanid (and delamanid, a different nitroimidazole that can be used in children) and there are no clinical studies investigating use in TBM (Table 3).

Another strategy is to combine bedaquiline with rifabutin, a rifamycin drug that, unlike rifampicin, does not result in clinically important increases in bedaquiline clearance^44,45^. Rifabutin has similar efficacy to rifampicin in (non-TBM) pre-clinical models and in observational patient cohorts^46^. Although there are no data on CNS penetration of rifabutin from TBM patients, several lines of evidence support potential efficacy in this condition. Rifabutin achieves high concentrations in CSF of healthy non-human primates^47^ (estimated free rifabutin CSF/plasma ratio 2.4–3.4), it is measurable in CSF of PWH^48^ and was effective in a rabbit model of pneumococcal meningitis^49^. Addition of rifabutin to a core regimen of bedaquiline plus pyrazinamide and moxifloxacin (BZM), both of which have excellent CNS penetration^35,50^, resulted in additive efficacy and had similar bactericidal and superior sterilising activity to standard therapy in a pulmonary tuberculosis mouse model experiment^51^. This regimen should be prioritised for clinical evaluation pending additional data confirming rifabutin exposure at site of disease in animal TBM models and CSF from tuberculosis patients.

### Future drug options

New compounds emerging from the pulmonary tuberculosis development pipeline may have a role in future TBM regimens. Sutezolid, a new generation oxazolidinone with a lower propensity for mitochondrial toxicity, is being evaluated as a replacement for linezolid in bedaquiline-based regimens for pulmonary tuberculosis. DprE1 inhibitors, offering a novel mechanism of action and potent antitubercular activity, are also in clinical development for pulmonary tuberculosis, including in combination with bedaquiline, pyrazinamide and moxifloxacin. Pharmacokinetic data need to be generated for sutezolid and DprE1 inhibitors before entering clinical trials for TBM. Another prospect is alpibectir (BVL-GSK098), a novel compound that increases bioactivation of ethionamide, requiring lower ethionamide doses to obtain rapid bactericidal activity when the two agents are combined (AlpE). AlpE is in active development for TBM based on a suite of favourable characteristics including good CNS penetration, activity against isoniazid resistant *M. tuberculosis*, improved tolerance, and limited drug-drug interactions, including with rifampicin. Ganfeborole (formerly GSK 3036656) targets *M. tuberculosis* leucyl-tRNA synthetase, inhibiting protein synthesis with rapid mycobacterial killing and sterilising ability^52^. Ganfeborole is not expected to be affected by drug-drug interactions with rifampicin, raising possibilities for combination with rifamycins and other novel antitubercular agents for TBM if CNS penetration is confirmed.

#### Host-directed therapy (HDT)

### General considerations

Interventions with anti-inflammatory effects are required to reduce immunopathology and consequent mortality and disability. There is rationale to investigate targeted therapies, directed at inflammatory molecules or pathways central to TBM pathophysiology that may complement or replace corticosteroids. The heterogeneity of inflammatory response between individuals suggest certain subgroups may derive more benefit from HDT than others. Examples include TBM in PWH, which is associated dysregulated inflammation, high mortality, and unclear benefit from corticosteroids; and patients developing paradoxical worsening during treatment who often require intensification of corticosteroid therapy. Identification of specific clinical phenotypes (for example, severity and nature of inflammation at baseline estimated by clinical, radiological or CSF markers) or genotypes (for example, variations in *leukotriene A4 hydrolase (LTA4H)* associated with distinct inflammatory phenotypes) that predict individual risk and treatment response is a priority. Mechanistic investigations nested in interventional trials can provide important insights. The LAST-ACT trial, which provides corticosteroids based on *LTA4H* genotype, is an example of targeted anti-inflammatory approach developed from translational studies in TBM^53^. However, until such time as other treatment-defining subgroups emerge, the priority is to evaluate an intervention that offers potential benefit to all patients, with possibility to identify subgroups for targeted intervention at a later stage.

### TNF-α antagonists

Tumour necrosis factor (TNF) is central to the immunopathology of TBM^54^. Retrospective case series data are promising for anti-TNF directed therapies of thalidomide and infliximab^55–59^. Initial enthusiasm for thalidomide was diminished after a trial among children with TBM in South Africa found an association with increased adverse effects and death when dosed at 24 mg/kg/day^60^. A more recent retrospective cohort study showed that much lower doses of 3-5 mg/kg/day thalidomide demonstrated satisfactory clinical and radiological response in 37/38 children with CNS tuberculosis-related complications^56^. However, there are ongoing barriers to thalidomide use for TBM including limited accessibility high cost, concerns about teratogenicity, and dose-related toxicity (e.g. neuropathy).

Specific TNF antagonists, particularly infliximab, are attractive options for definitive evaluation in TBM trials. A large case series provides strong preliminary support for safety and efficacy in TBM^58^, and there is accumulating clinical experience with use for paradoxical reactions in CNS TB. Infliximab is widely used for other inflammatory conditions with established safety in adults and children^61–63^ and no signal of major infection complications, including inflammatory bowel disease where there is high risk of bacterial translocation. Increased risk of tuberculosis is a lesser concern as all individuals will receive antitubercular chemotherapy. Cost is not expected to be a major limitation as generic preparations are now available, although consideration must be given to availability of, and access to, infliximab or biosimilars after the trial. The optimal dose and number of infusions of infliximab for TBM remains uncertain and pharmacometric data would help to optimise use. An additional event-driven randomisation to a second dose of infliximab (or another host directed therapy) for a subgroup with neuro-deterioration with inflammatory complications may be considered. This decision could be based on the performance of infliximab when provided to all participants at study entry (if successful it may substantially reduce delayed complications and reduce the need for a second randomisation).

### Other considerations for host directed therapy

Detailed investigations of TBM immunopathology from phase 2 trials, observational studies, and animal models can generate alternatives to corticosteroids (Table 4). Small trials suggest that adjunctive aspirin may provide safe and beneficial anti-inflammatory effects in children and adults with TBM^64–67^, supporting ongoing phase 3 (Table 1).

Immunopathological studies have implicated multiple cytokines in TBM pathogenesis^68,69^. Use of the IL-1 receptor antagonist anakinra has been described in both PWH and TBM and HIV-negative TBM^70^. However, costs and accessibility currently preclude use in low-resource settings, and a need for daily intravenous or subcutaneous administration present challenges. Other immunomodulatory agents may offer potential benefit, such as JAK-inhibitors (e.g. baricitinib) which have broader anti-inflammatory activity and good safety profile, however, their evaluation in any form of tuberculosis hitherto has been very limited.

## Evaluating therapies in a global trial

### Key study populations

TBM affects all age groups but is especially common amongst young children and in PWH. Therefore, therapeutic trials, particularly phase 3 trials, should include these patient groups. It is also essential that all disease severities are included in future trials. Some previous trials of corticosteroids excluded those with mild disease (MRC grade 1), believing inflammation, and therefore likely benefit, was less in these patients^27^. However, the 2004 Vietnam trial showed that whilst dexamethasone benefited all three MRC severity grades, and sustained benefit beyond 2 years was only seen in those with grade 1 disease^71^. Conversely, there is risk of systematic exclusion of patients with more severe disease without capacity to provide informed consent themselves, and ethics committees should support mechanisms for obtaining surrogate consent to provide the sickest patients an opportunity for trial participation.

The limited sensitivity of current diagnostic tests for TBM means ascertaining the true population with TBM is difficult. In 2010, the TBM consortium published a uniform case definition for TBM that is now widely used to categorise TBM research participants into definite, probable, possible, and not TBM^72^. This case classification is applied retrospectively after all diagnostic information has returned thus is not practical for eligibility at enrolment. Enrolling cases of suspected TBM, based on clinician intention to treat for TBM, is the most pragmatic approach for phase 3 trials and reflects real-world clinical practice. However, this may result in enrolment of cases eventually re-classified with a different diagnosis. Cases of possible TBM who are treated for TBM are heterogenous across different settings, with approaches to commencing antitubercular chemotherapy particularly influenced by the HIV and tuberculosis prevalence in that population. Therefore, increased sample sizes might be required recognising that a small proportion might not have TBM and therefore may respond differently to new interventions.

### Sites and countries

TBM is a disease of poverty. It is commonest in settings least able to deliver the clinical care and research required to reduce its frequently fatal consequences. TBM research must therefore promote and expand research capacity in less well-resourced or developed centres, building a sustainable global infrastructure and community capable of performing high-quality clinical research. There is a core of centres, developed over the last 20 years, that now have established track records of performing TBM trials, in India, Indonesia, Madagascar, South Africa, Uganda, and Vietnam. To date, they have tended to conduct their trials independently. Coordination and collaboration between these centres could enable annual trial recruitment rates of around 1000 participants/year, which would have a dramatic effect on the speed and power of future TBM trials.

### Trial design

For some drugs and regimens, further evidence is required from phase 2 evaluation of safety and PK before they can enter practice-defining phase 3 trials. Phase 2 trials can also be exploited for mechanistic investigation that may identify targets for immune modulation and translational evaluation. However, in the absence of predictive treatment response biomarkers, phase 2 trials are unable to provide actionable information on efficacy because they are underpowered for disability and mortality, the only available efficacy measures in TBM. The current approach, where independent centres conduct small and sequential phase 2 then 3 trials of single interventions leads to decades-long delays before new drugs or regimens benefit patients. In this context, phase 3 evaluation of promising interventions may be justifiable without phase 2 trials with demonstrable site of disease exposure from preclinical models and safety data from PTB.

A global multi-arm, potentially multi-stage, factorially randomised (antitubercular chemotherapy and anti-inflammatory drugs), controlled trial could address many of the current obstacles to improving outcomes from TBM. A platform trial offers the ability to study different interventions in parallel and to introduce new interventions over time, including at site level based on accessibility and other local considerations. Having a master protocol agnostic to interventions studied with the ability to add a next intervention could also maximise overall trial impact, if one intervention ends early (for superiority or harm). This design has much appeal but introduces funding challenges that need further exploration with relevant agencies.

### Statistical considerations

A large trial is needed to demonstrate superiority of new antitubercular and anti-inflammatory regimens over standard of care. Assuming typical mortality, a trial of 900 patients per arm would be required to detect a 20% reduction in mortality with 90% power. Making no presumption of interaction between antitubercular and anti-inflammatory drugs, an efficient design is factorial randomization across two domains:

Domain 1: Antitubercular therapy. The trial would ideally investigate at least three regimens, with one of them being a standard-of-care regimen (following WHO recommendations). For practical reasons (rifampicin stains secretions red) this randomisation would likely be open label, but outcome assessors would be blinded.

Domain 2: HDT. The priority intervention is infliximab versus standard of care (corticosteroids). Infliximab would be combined with corticosteroids in the experimental groups. This randomisation could be blinded with a placebo saline infusion. As the efficacy of HDT regimens may differ between people with and without HIV, a basket trial design could be adopted with partial pooling across the two groups.

### Outcomes

The TBM consortium have published two consensus statements (2017 and 2019) concerning standardised methods for enhancing the quality and comparability of TBM studies, with recommendations for primary and secondary outcomes for phase 2 and 3 TBM clinical trials (Table 5)^12,15^. Improving disability-free survival is the primary objective of successful TBM treatment. Thus, a composite endpoint of death and severe disability at 1 year from randomisation is recommended for phase 3 TBM trials. The Modified Rankin Score (MRS) is widely used to assess functional disability after stroke and has been used in many recent TBM trials^73^. Choosing an appropriate MRS cut-off to define disability is complicated by differing cultural perceptions and consequences of disability, often related to the resources available for the long-term care of disabled individuals. Patient and community stakeholder engagement is needed to identify culturally appropriate outcomes that are desired by persons afflicted with TBM. Health economic endpoints (DALY, QALY) should be acquired to understand the impacts of new interventions on individuals and society and provide essential information for policy makers.

Important secondary outcomes include the occurrence of the common intracerebral (e.g. stroke) and extracerebral (e.g. hyponatraemia) disease complications. Longer-term cognitive impairment is well recognised, especially in children, but poorly studied, partly because of the complexity of assessment methods^15^. Cognitive assessments may therefore only be possible in selected centres, but they represent an essential substudy.

Some pragmatism is justified to support delivery of a global phase 3 trial, particularly if the safety profile of the intervention is well-known. Safety reporting should focus on serious treatment-related adverse events rather than those related to the severity and complications of the disease.

### Governance and sponsorship

Models of sponsorship and governance should reflect the importance of keeping the centre of gravity of TBM research within low- and middle-income countries (LMIC), thus enabling local decision-making and building capacity, expertise and leadership for future research. Engagement with industry will be essential, given the need to test new drugs. Trials will therefore need to meet the regulatory standards of conduct, necessary to allow global approval of new drugs for TBM treatment. This will undoubtedly be challenging but forms an essential part of building a global infrastructure to conduct TBM trials. Given the need to test multiple drugs, potentially from different companies, an umbrella sponsorship model will be required, ideally from an LMIC-based academic institution. The role for contract research organisations is anticipated to be small, as they are perceived to substantially increase cost and complexity, to excessively emphasize regulatory compliance above operational efficiency, and fail to build local trial infrastructure and expertise for future trials.

## Conclusions and future directions

Only adequately powered, definitive trials with clinical endpoints that are relevant to patients and their carers can address the unacceptable outcomes in TBM. This goal requires a recognition from funders that it is not reasonable to neglect the most serious form of tuberculosis on the grounds that it does not contribute to transmission and thus global elimination targets. To do so marginalises already vulnerable populations in which this disease is common and misses opportunity to improve overall treatment of tuberculosis, with enormous potential benefits to individuals and communities. EndTB targets – 90% reduction in deaths by 2030 - can only be achieved if the most severe forms of TB are tackled. Efficient and pragmatic clinical trials, presented in this personal view, would evaluate several readily implementable interventions for communities most affected by TBM in a cost-effective manner. This represents an opportunity for funders to make investments with direct and lasting benefit for many people in LMIC. Risk could be mitigated through seamless phase 2/3 evaluation of novel therapies and with innovative funding models that involve multiple stakeholders, including drug manufacturers. The Tuberculous Meningitis International Research Consortium will continue working towards improving treatment for people with TBM through high quality clinical trials.

## Figures and Tables

**Table 1:** Current and planned clinical trials in tuberculous meningitis

#	Trial	N	Enrolment Status	Estimated Completion	Registration	Population	Intervention
**1**	ACT TBM	237	100%	Complete	CTRI/2019/08/020488	Adults	ASA or CLOPI or standard care
**2**	SIMPLE	36	100%	Complete	NCT03537495	Adult	High dose RIF + LZD
**3**	ALTER	40	100%	Complete, unpublished	NCT04021121	Adults	High or standard dose RIF + LZD
**4**	LAST ACT	720	100%	Mar 2024	NCT03100786	HIV- Adults	Corticosteroids stratified by *LTA4H* genotype
**5**	HARVEST	500	53%	Nov 2024	ISRCTN15668391	Adults	High dose RIF
**6**	INTENSE TBM	768	43%	Aug 2025	NCT04145258	Adults, >15yo	High dose RIF+LZD +/-ASA
**7**	TIMPANI	130	0%	Dec 2025	NCT05590455	HIV+ Adults	Adalimumab (TNF inhibitor)
**8**	SURE	400	80%	Dec 2025	ISRCTN40829906	Children + adolescents	High dose RIF and INH + LFX w/ or w/o ASA
**9**	IMAGINE TBM	330	5%	Feb 2027	NCT05383742	Adults, >15yo	High dose RIF and INH + LZD x 6 mos.
**10**	INSHORT	372	0%	Sept 2027	NCT05917340	Adults	High dose RIF + moxifloxacin + ASA x 6 mos.

Short form titles or descriptions: ACT TBM = A Randomised Trial to Assess the efficacy or add on therapy with Aspirin or Clopidogrel to the standard medical therapy alone in patients with Tuberculous meningitis. SIMPLE = Pharmacokinetic Study of Linezolid for TB Meningitis. ALTER = Adjunctive Linezolid for the Treatment of Tuberculous Meningitis. LAST ACT = Leukotriene A4 Hydrolase Stratified Trial of Adjunctive Corticosteroids for HIV-uninfected Adults with Tuberculous Meningitis. TIMPANI = TNF Inhibitors to Reduce Mortality in HIV-1 Infected Patients with Tuberculous meningitis. HARVEST = High-dose oral rifampicin to improve survival from adult tuberculous meningitis. INTENSE-TBM = Intensified Tuberculosis Treatment to Reduce the Mortality of Patients with Tuberculous Meningitis. SURE = Short intensive treatment for children with tuberculous meningitis. IMAGINE-TBM = Improved Management with Antimicrobial Agents Isoniazid Rifampicin Linezolid for TBM. HDH Trial = Optimizing Antituberculosis Therapy in Adults with Tuberculous Meningitis. ASA=aspirin. CLOPI=clopidogrel. HIV=human immunodeficiency virus. INH=isoniazid. *LTA4H=leukotriene A4 hydrolase*. LFX=levofloxacin. LZD=linezolid. NAT-2. N-acetyltransferease-2. RIF=rifampicin. TNF=tumour necrosis factor.

**Table 2:** A scoring system to select drugs for inclusion in experimental regimens

	Anti-TB activity	Clinical efficacy data in TBM	Site of disease exposure	Safety/tolerability	Drug-drug interactions	Access
**3**	Potent bactericidal sterilising activity *in vivo*		Potentially therapeutic concentrations in brain parenchyma from animal models and/or non-invasive human studies at human-equivalent doses			
**2**	Moderate *in vivo* activity, mainly related to EBA	Benefit in RCT	Detectable concentrations in brain parenchyma but possibly below therapeutic thresholds	Well- tolerated at optimised doses with low toxicity potential		Affordable and available in target countries, oral administration
**1**	Weak *in vivo* activity at tolerable doses	Benefit in non- randomised studies, sub- groups in RCT, or case reports/series	Detectable in CSF only or not yet studied in brain parenchyma	Generally well-tolerated but may have treatment- limiting AE	No clinically relevant DDI	Affordable and available in target countries, IV administration
**0**	*In vitro* data only	No data from trials or case reports	Insufficient / no data to judge	Poorly tolerated but acceptable safety profile	Clinically important DDI	Expensive and/or not registered in target countries
**No go**	No *in vivo* activity (EBA) attolerable doses	RCT data shows no effect	Undetectable in brain parenchyma at human- equivalent doses	Poorly tolerated and/or frequent treatment- limiting AE	Precludes use in TBM regimens	Not currently manufactured

Individual drugs are ranked by adding points from each parameter; the higher number of points the higher the priority for inclusion in experimental TBM regimens. Extra weighting is applied to anti-TB activity and site of disease exposure by creating categories for higher point allocation. Lower weighting is applied to clinical efficacy in TBM because new agents are less likely to have been evaluated in clinical trials. Similarly, safety/tolerability is weighted less because drug efficacy is prioritised for this condition with high early mortality. Drug-drug interactions and access are assigned fewer available points because of limited categories. AE=adverse events. DDI=drug-drug interactions. EBA=early bactericidal activity. IV=intravenous. RCT=randomised controlled trial. TBM=tuberculous meningitis.

**Table 3:** Characteristics of individual drugs for use in TBM

	Anti-TB activity	Clinical efficacy in TBM	Site of disease exposure	Safety/ tolerability	Drug-drug interactions	Access
**Rifampicin**	3	2	3	2	0	2
**Isoniazid**	3	2	3	2	1	2
**Linezolid** ^74^	2	2	3	1	1	2
**Fluoroquinolones** ^50^	3	2	3	2	1	2
**Pyrazinamide**	2	2	3	2	1	2
**Pretomanid/delamanid** ^75^	3	1	3	2	1	2
**Bedaquiline**	3	1	2	2	0	2
**Alpibectir/ethionamide**	2	0	3	2	1	
**Rifabutin**	3	0	2	2	0	2
**Clofazimine**	1	0	1	2	1	2
**Ethionamide**	1	2	2		1	2
**Cycloserine**	1	0	2		1	2
**Ethambutol**	1			2	1	2
**DprE1 inhibitors**	3	0	0	2	1	

Scores derived from the scoring system in Table 2.

**Table 4:** Host directed therapies for TBM

	Activity in TBM	Clinical use	CNS exposure	Safety
**Corticosteroids**	**Large RCTs: Adults: 25% lower mortality ^3^, smaller effect for MRC grade 2/3 and with longer follow up ^71^; no effect on disability; uncertain effect in HIV ^26^. Scarce data among African adults and Asian paediatric TBM**	**Guideline- recommended for all patients with TBM, including IRIS and paradoxical reactions**	**Good**	**Excellent in** **TBM RCT ^76^**
**Aspirin**	**Small RCT: Possibly fewer new-onset strokes at high doses among adults with TBM ^65^**	**Not in routine clinical use, evaluated in adults and children with new TBM diagnosis**	**Good**	**No signal of severe bleeding events ^77^**
**Thalidomide**	**Individual case reports of resolution from mass lesions and blindness related to optochiasmatic arachnoiditis (children)**	**Steroid-refractory TBM or paradoxical reactions**	**Good**	**Dose related toxicity, paediatric RCT stopped prematurely for safety ^60^**
**TNF blockers (infliximab)**	**Case series ^57,59,78^ and matched retrospective cohort ^58^ showing clinical benefit in TBM**	**Steroid-refractory TBM or paradoxical reactions**	**Good**	**No serious safety signals, Risk of secondary infection**
**Anti-IL1** **(anakinra)**	**Case reports in TBM ^70,79^**	**Steroid-refractory TBM or paradoxical reactions**	**Good**	**Good safety profile, associated with mild neutropenia**
**mTOR inhibitors**	**RCT: Less post-TB lung disease**	**No experience in TBM**	**Unknown**	**Well tolerated in an RCT for PTB**
**JAKi**	**Cases reports for HLH /** **HLH-TB ^80^**	**No experience in TBM**		**Good safety profile, associated with VZV/HSV**

**Table 5:** Outcomes according to phase

Phase	Primary endpoint(s)	Secondary endpoint(s)
II	Adverse events of special interest (AESI) Serious adverse events (SAE) Pharmacokinetic analyses	Drug-drug interactions Treatment interruption (tolerability) Mortality and disability Biomarkers of treatment response (pathogen and host inflammation)
III	Mortality^a^ at 12 mo. Disability by MRS at 12 mo.	Change in GCS or MRC grade Change in neuroimaging Occurrence of new events Occurrence of paradoxical deterioration or TBM-IRIS Cognitive status at 12 mo. SAE with grade and relatedness Duration of hospitalisation AESI Treatment interruption (with reason and duration) Health Economic (improvement in DALY and QALY)

a All cause and attributable to TBM

DALY = Disability adjusted life year(s)

EOT = End of treatment

GCS = Glasgow coma scale

MRS = Modified Rankin scale

QALY = Quality adjusted life year(s)

TBM-IRIS = HIV-tuberculosis associated immune reconstitution inflammatory syndrome Table based on ^12,15^.
